# Endocrine-Disruptive Effects of Adenylate Cyclase Activator Forskolin: In Vitro and In Vivo Evidence

**DOI:** 10.3390/toxics12100701

**Published:** 2024-09-27

**Authors:** Chong Huang, Yanbin Zhao, Jianying Hu

**Affiliations:** 1 MOE Laboratory for Earth Surface Processes, College of Urban and Environmental Sciences, Peking University, Beijing 100871, China; hc@urban.pku.edu.cn (C.H.); hujy@urban.pku.edu.cn (J.H.); 2 State Environmental Protection Key Laboratory of Environmental Health Impact Assessment of Emerging Contaminants, School of Environmental Science and Engineering, Shanghai Jiao Tong University, Shanghai 200240, China

**Keywords:** forskolin, endocrine disruption, steroidogenesis, H295R, medaka

## Abstract

Forskolin (FSK) is a potent adenylate cyclase activator and may display endocrine-disruptive effects via the disruption of steroidogenesis. Here, we tested this hypothesis by use of the in vitro H295R steroidogenesis assay and the in vivo long-term medaka (*Oryzias latipes*) exposure assay. The results from the H295R assay demonstrated that the transcriptional levels of a series of genes involved in steroidogenesis, including *HSD3B2*, *CYP11A*, *CYP11B2*, *CYP17*, *CYP19*, and *CYP21*, were remarkably up-regulated. Meanwhile, the productions of estrogens (17β-estradiol (17β-E_2_) and estrone (E_1_)) and progestins (progesterone (PGT) and 17-hydroxyprogesterone (17-HPT)) were significantly increased, and those of androgens (androstenedione (ADD) and testosterone (TTR)) were significantly inhibited. After waterborne exposure of medaka to FSK for 100 days, the gene expressions of *HMGR*, *HSD17B1*, *CYP17B*, *CYP19A*, and *CYP21A* were significantly enhanced in the gonads of male medaka. 17β-E2 was remarkably induced, although without statistical significance. In addition, the biomarker genes for estrogenicity, including *VTG-I*, *VTG-II*, *CHG-H*, and *CHG-L*, were significantly induced in male medaka livers. Pathological damage to their gonads was further identified. Therefore, the results demonstrated that FSK modulates the transcriptions of steroidogenesis genes and alters hormone levels in vitro and in vivo, which is a mark of endocrine disruption in organisms.

## 1. Introduction

Endocrine disrupting chemicals (EDCs) can interfere with the endocrine system and cause a series of adverse effects on endocrine homeostasis, development, and even reproduction in vertebrates as well as humans [1,2]. They exert their effects either by binding to hormone receptors, leading to activation or inhibition of its signaling pathway, or by affecting endogenous hormone concentration or its availability, or by modifying hormone receptor turn over [3]. Among the EDCs, significant attention has been focused on substances that exhibit binding affinity to receptors, particularly nuclear receptors such as estrogen receptors and thyroid hormone receptors. These substances exert biological effects by mimicking endogenous hormones and activating the associated receptor signaling pathways [4,5]. On the contrary, substances with other mechanisms of action, such as the disruption of endogenous synthesis processes of hormones and the consequent modulation of hormone production within the organism [6], have not garnered comparable attention.

Cytochrome P450 aromatase (*CYP19*) is a pivotal steroidogenic enzyme, responsible for the conversion of C19 androgens to C18 estrogens in vertebrates. As such, aromatase activity is an important modulator of 17β-estradiol (E_2_) concentrations and is critical for the regulation of processes governed by E_2_. Recently, a series of environmental substances, such as the herbicide atrazine and the antimycotic drug clotrimazole, have been identified as EDCs targeting aromatase activity [7,8]. Notably, in addition to aromatase, a suite of cytochrome P450 enzymes participating in steroidogenesis, like *CYP11*, *CYP17*, and *HSD17B1*, may also serve as targets for environmental substances [9,10] and undergo very limited explorations.

Forskolin (FSK), a cell-permeable diterpene that directly activates adenylyl cyclase, has found widespread application as a pharmaceutical drug for treating cardiovascular diseases such as hypertension and angina, as well as respiratory disorders like asthma [11]. FSK has been shown to stimulate adenylate cyclase activity, resulting in an increase in intracellular cAMP levels. This increase, in turn, activates cAMP-dependent protein kinase and other cAMP receptor proteins [12]. As a second messenger, cAMP plays a crucial role in cell signaling, regulating numerous physiological and pathological processes [13]. cAMP regulates the transcription of various target genes, including those involved in PKA, Epac1, Epac2, and nucleotide-gated ion channels [14,15]. Its effects are primarily mediated through PKA and its downstream effectors, such as cAMP-responsive element-binding protein (CREB) [13,16]. Additionally, PKA can phosphorylate various kinases, including Raf, GSK3, and FAK. The disruptions in cAMP–PKA signaling are implicated in various types the of human tumors [17]. Consequently, FSK can act as a relatively non-specific activator of cytochrome P450 enzymes (CYPs), thereby enhancing the activity of a broad spectrum of CYP enzymes in vertebrates [18]. However, whether FSK can activate CYPs participating in steroidogenesis and consequently exhibit endocrine disruptions remains an area that has been minimally explored. In recent studies, forskolin has been shown to stimulate cAMP-mediated acute steroidogenesis and likely up-regulate the transcriptional levels of aromatase in vitro in a dose-dependent manner [19,20]. A general increase in the production of 17β-estradiol has also been reported for cells upon FSK exposure [21]. Therefore, these outcomes provided a compelling ground for undertaking a comprehensive exploration of the endocrine-disruptive effects of FSK.

On this basis, the aim of the present study was to evaluate the potential endocrine-disruptive effects of FSK. To achieve this, we employed the in vitro H295R steroidogenesis bioassay and the long-term medaka (*Oryzias latipes*) exposure assay. The H295R steroidogenesis assay was developed to quantitatively assess the effects of xenobiotics on the transcription of genes involved in steroidogenesis [21,22]. H295R cells express key enzymes essential for steroid hormone synthesis, and this assay has been widely and successfully applied to characterize the impacts of chemicals on steroidogenic processes. In addition to the measurements on the genes participating in steroidogenesis and the production of steroid hormones, multiple developmental and reproductive endpoints of medaka, including reproductive success, the hepatic-somatic index, the histopathology of gonads, the fertilization success of F1 eggs, as well as the changes in biomarker genes for estrogenicity, were determined. The present analysis aims to provide novel insights into the toxicological effects of FSK, thereby contributing a novel perspective to investigate EDCs.

## 2. Materials and Methods

**Chemicals and Reagents.** Forskolin (purity: ≥98%) was purchased from Apollo Scientific Ltd. (Stockport, Cheshire, UK and were dissolved in dimethyl sulfoxide (DMSO) as the stock solution stored at −20 °C. All chemicals and reagents used in this study were of molecular biology grade unless otherwise described. E1 and 17β-E2 were purchased from Wako (Tokyo, Japan). PGT, 17-HPT, TTR, ADD, E1, and 17β-E2 were purchased from Sigma-Aldrich (St. Louis, MO, USA). PGT-d_9_, 17β-E2-d_3_, E1-d_2_, and TTR-^13^C_2_ were purchased as powders from Wako (Tokyo, Japan). Methanol, acetone, ethyl acetate, hexane, diethyl ether, and dichloromethane were all HPLC-grade, obtained from JT Baker (Phillipsburg, NJ, USA).

**In Vitro H295R Cell Assay.** H295R cell line was obtained from the American Type Culture Collection (ATCC CRL-2128; Manassas, VA, USA), and the cells were cultured as described in our previous paper [23]. Cells were seeded at a density of approximately 1 × 10^6^ cells/mL per well in a six-well cell culture plate. After growth for at least 24 h, the culture medium was removed, and cells were exposed to 2 mL of dosing solution at four serial concentrations of FSK (0.3 μM, 1 μM, 3 μM, 30 μM) for 48 h. The concentration range was selected based on the results of our preliminary experiments. The concentration of DMSO in each test was kept constant at 0.1%. All treatments were conducted in triplicate. After 48 h of exposure, the cell culture supernatant collected from exposure wells was collected and stored at −80 °C for hormone analysis. The adherent cells in each well of 6-well plates were used for isolation of total RNA using Trizol reagent (Invitrogen Life Technologies, Carlsbad, CA, USA). Nucleic acid isolation, expression analysis of target genes by RT-PCR, and hormone analysis by UPLC/MS-MS were performed as we described previously [23].

**In Vivo Medaka Exposure.** Eggs were collected from female medaka from a breeding stock of adult fish, as described previously [24]. They were pooled in embryo-rearing medium in a petri plate and checked for fertilization. Afterwards, eggs were raised to hatch, and exposure to test chemicals was initiated during early life stages of medaka at 1 d after hatch in a static-renewal system. Water employed in the experiment was active carbon-treated with a hardness of 81.1 ± 1.2 mg L^−1^ CaCO_3_, pH of 7.7 ± 0.2, and dissolved oxygen of 7.8 ± 0.3 mg L^−1^ at 25 ± 1 °C. Exposures took place in glass containers of progressively larger sizes (i.e., 2 and 12 L) as the medaka grew. We prepared stock solutions of FSK in DMSO and administered the stock solutions into exposure systems at a ratio of 100 μL of stock solution per liter of water. The concentrations for FSK were set at 0.03, 0.3, 3, and 30 μg/L. The concentration range was selected based on the results of our preliminary experiments. The aqueous solutions in the test containers were renewed every 24 h during the exposure period. Each treatment included 60 fish at the start of the experiment. The fish were maintained in a light/dark cycle of 16:8 h and were fed a diet of newly hatched brine shrimp twice daily. During the final exposure week, reproduction and larval development experiments were carried out. Spawned eggs were collected daily at 2–3 h after fertilization. Mean numbers of spawned eggs were calculated. Larval development was examined using a Leica M165 FC (Made in Singapore). Larvae with deformations were identified and recorded. After the exposure, medaka fish were euthanized with tricaine methane sulfonate (MS-222). Phenotypic sex of individual medaka was assessed by examining externally visible secondary sex characteristics under a dissecting microscope. Then, six males were sampled from each group for gene expression analysis of the liver and gonads, which were frozen in liquid nitrogen until the RNA was isolated.

**Gene Expression Analysis.** RNA extraction and qRT-PCR analysis were performed according to the methods we described previously [21]. In brief, total RNA was extracted using TRIzol Reagent (Gibco BRL, Life Technologies, Gaithersburg, MA, USA), and cDNA was synthesized using TaqMan Reverse Transcription Reagents (Applied Biosystems, Foster City, CA, USA). Quantitative real-time PCR (qRT-PCR) was conducted with SYBR Mix (Applied Biosystems, Foster City, CA, USA) on an ABI Prism 7000 Sequence Detection System (Applied Biosystems, Foster City, CA, USA). PCR primers used for the quantification are shown in Table S1 (for H295R) and Table S2 (for medaka); β-actin and RPL-7 (ribosomal protein L7) were used as internal controls [21]. The normalized qRT-PCR results were analyzed using the 2^−ΔΔCt^ method.

**Hormone Analysis of Exposed Fish.** Concentrations of steroid hormones were analyzed by ultra-performance liquid chromatography–tandem mass spectrometry (UPLC-MS/MS). This method for estrogen analysis of the whole fish was described previously [25]. In this study, additional steps were taken for androgen analysis during the previous extraction process as follows: After the silica cartridges were rinsed with 3 mL of hexane/ethyl acetate (90:10, *v*/*v*), the fraction containing estrogens and androgens were eluted with 3 mL of hexane/ethyl acetate (38:62, *v*/*v*). The elution was dried and redissolved with 2 mL of hexane-methylene chloride (DCM) (1:1, *v*/*v*); 1mL of the elution was evaporated to dryness under a gentle stream of nitrogen and reconstituted with 0.2 mL of methanol for androgen analysis by UPLC-MS/MS. The remaining 1 mL of the elution was then passed through preconditioned Florisil cartridges (6 mL, 1 g) obtained from Waters (Milford, MA, USA). total of 10 mL of a mixture of 76 hexane-DCM (1:1, *v*/*v*) was discarded, and the fraction containing all estrogens was eluted with 6 mL of acetone-DCM (1:9, *v*/*v*). The solution was evaporated to dryness under a gentle stream of nitrogen and reconstituted with 0.2 mL of methanol for UPLC-MS/MS analysis.

**Histopathological Analysis.** The gonads of male medaka were sampled, fixed in 10% neutral buffered formalin for more than 24 h, and then dehydrated in a graded series of ethanol solutions embedded in paraffin blocks according to standard methods. Sections were cut at 5–10 μm and stained with hematoxylin and eosin. Slides were examined by a Leica M165 FC (Made in Singapore) (Leica M165 FC, Wetzlar, Germany) for histopathological analysis.

**Statistical Procedures.** Gene expressions and hormone levels were calculated as fold change relative to those of the solvent control. SPSS 11.5 (Applied Bioscience, Foster City, CA, USA) was used to analyze all the collected data. Before using parametric statistics, the normality of each sample set was assessed with the Kolmogorov–Smirnov one-sample test with Lillifor’s transformation. Variance homogeneity was determined with Levene’s test. Differences between groups were assessed using one-way analysis of variance (ANOVA), followed by Tukey’s test. Differences with *p* < 0.05 were considered significant.

## 3. Results and Discussion

**Figure 1:**
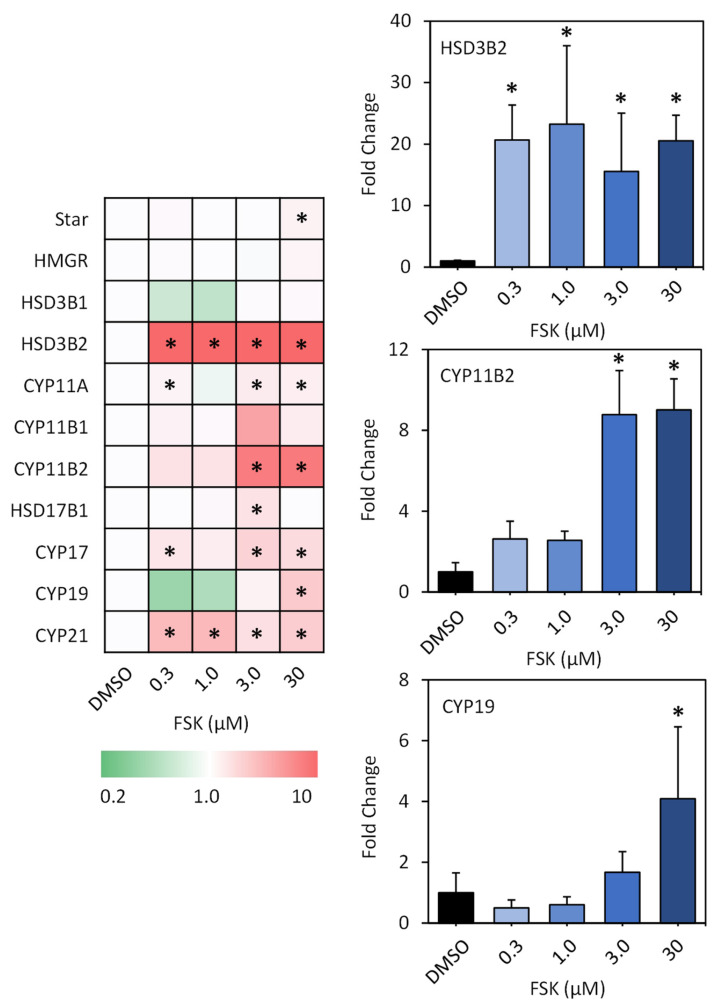
Alterations in steroidogenesis-related genes in H295R cells exposed to forskolin for 48 h. Key for colors: red: up-regulation; green: down-regulation. The alterations in the representative genes (CYP11B2 and CYP19) are presented on the right. Data are expressed as fold change relative to the solvent control (*n* = 3). Asterisk (*) indicates statistically different from the control (*p* < 0.05).

**Gene Expressions in H295R Cells.** H295R cells were exposed to different concentrations of forskolin (0.3 μM, 1 μM, 3 μM, and 30 μM) for 48 h. As shown in Figure 1 and Table S3, a series of genes involved in steroidogenesis were significantly up-regulated, including *StAR*, *HSD3B2*, *CYP11A*, *CYP11B2*, *CYP17*, *CYP19*, and *CYP21*. Among them, *HSD3B2* was the most severely affected. The transcriptional levels were induced by 20.7, 23.2, 15.6, and 20.5 times (fold change) in the cells upon 0.3 μM, 1.0 μM, 3 μM, and 30 μM FSK exposure, respectively. Following this, *CYP21* exhibited induction in transcriptional levels by 5.0, 5.2, 2.8, and 3.9 times in the cells upon exposure to 0.3 μM, 1.0 μM, 3 μM, and 30 μM FSK, respectively. It should be noted that *CYP11B2* and *CYP19* were also significantly up-regulated, although not at low concentrations. Fold changes by 9.0 and 4.1 times were demonstrated for *CYP11B2* and *CYP19* at an FSK concentration of 30 μM, respectively (Figure 1).

*HSD3B2* encodes a bifunctional enzyme that facilitates the oxidative conversion of delta(5)-ene-3-beta-hydroxy steroids and ketosteroids. It is predominantly expressed in the adrenals and gonads, playing a crucial role in the biosynthesis of all classes of steroids [26]. In comparison, *CYP11B2*, *CYP19*, and *CYP21* are downstream enzymes in the steroidogenesis process. *CYP11B2* is responsible for catalyzing the biosynthesis of aldosterone, the primary mineralocorticoid in vertebrates responsible for salt and water homeostasis, thereby influencing blood pressure regulation and the development of heart failure [27]. *CYP19* is monooxygenase that catalyzes the conversion of C19 androgens to C18 estrogens, such as estrone and estradiol [28]. *CYP21* catalyzes the hydroxylation at C-21 of progesterone and 17alpha-hydroxyprogesterone, leading to the formation of 11-deoxycorticosterone and 11-deoxycortisol, intermediate metabolites in the biosynthetic pathway of mineralocorticoids and glucocorticoids [29]. Thereby, the significant alterations in steroidogenesis genes such as *HSD3B2*, *CYP11B2*, and *CYP19* suggest that substantial changes in steroid hormone levels may occur. Furthermore, it is important to note that forskolin has been shown to stimulate steroidogenesis through cAMP signaling and to up-regulate the transcriptional levels of aromatase in vitro [19,20]. Thus, the present study suggests that forskolin may target multiple steroidogenesis genes, including *HSD3B2* and *CYP11B2*, in addition to aromatase, warranting further investigation.

**Figure 2:**
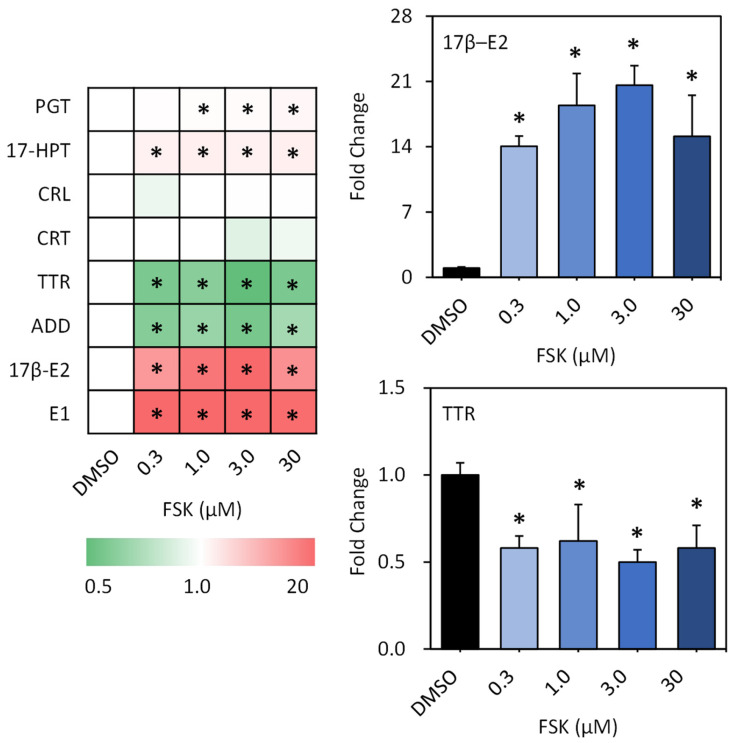
Alterations in steroid hormone production in H295R cells exposed to forskolin for 48 h. Key for colors: red: up-regulation; green: down-regulation. The alterations in representative steroid hormones (17β–E2 and TTR) are presented on the right. Data are expressed as fold change relative to the solvent control (*n* = 3). Asterisk (*) indicates statistically different from the control (*p* < 0.05).

**Hormone Production in H295R Cells.** Steroid hormones including 17β-E_2_, E_1_, TTR, ADD, PGT, 17-HPT, cortisol (CRL), and corticosterone (CRT) were further measured to explore the adverse effects of FSK on hormone production in H295R cells. As shown in Figure 2 and Table S4, we observed significant alterations for six steroids including 17β-E_2_, E_1_, TTR, ADD, PGT, and 17-HPT. Meanwhile, negligible effects were observed for CRL and CRT. Among them, 17β-E_2_ and E_1_ were the most severely affected. Their concentrations increased by 14.7, 18.4, 20.6, and 15.1 times, and 22.9, 31.6, 27.0, and 19.5 times in the cells upon 0.3 μM, 1.0 μM, 3 μM, and 30 μM FSK exposure, respectively (Figure 2). Following this, 17-HPT exhibited remarkable inductions in its concentrations by 2.9, 3.1, 2.8, and 3.2 times in the cells upon exposure to 0.3 μM, 1.0 μM, 3 μM, and 30 μM FSK, respectively. Meanwhile, dose-dependent increases in PGT concentrations were also observed. Significant inductions by 1.5, 1.6, and 2.1 times in cells upon exposure to 1 μM, 3 μM, and 30 μM FSK, respectively, were observed. In comparison, TTR and ADD were slightly inhibited. Their concentrations decreased by 0.6, 0.6, 0.5, and 0.6 times, and 0.6, 0.7, 0.6, and 0.7 times in cells upon 0.3 μM, 1.0 μM, 3 μM, and 30 μM FSK exposure, respectively (Figure 2). Therefore, these results were consistent with the outcomes observed at the transcriptional level, such as the significant alterations in the *CYP11B2*, *CYP17*, *CYP19*, and *CYP21* levels, suggesting that hormonal homeostasis was disrupted. Steroids play an indispensable role in maintaining the balance of the endocrine system. Disruptions in steroid levels can distinctly contribute to endocrine system disorders. For example, 17β-E_2_ plays a crucial role in regulating women’s reproductive cycles, influencing the development of secondary sexual characteristics in women, as well as the maintenance of reproductive tissues such as mammary glands and the uterus. Disruptions in 17β-E_2_ are implicated in a range of endocrine diseases, including polycystic ovarian syndrome and breast cancer [30]. Consequently, the present in vitro tests suggest a potent endocrine-disruptive effect of FSK in cells, with potential implications for similar effects in vivo.

**Figure 3:**
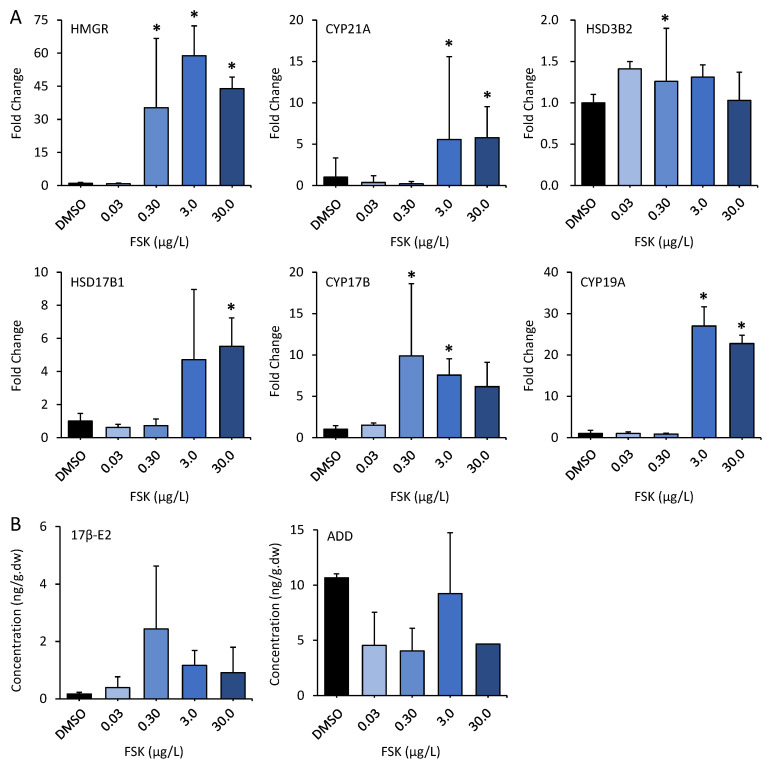
Fold changes of expressions of steroidogenesis-related genes (**A**) and the production of steroid hormones (**B**) in male medaka after 100-day forskolin exposure (*n* = 6). Asterisk (*) indicates statistically significant differences from the control (*p* < 0.05).

**Gene Expressions and Hormone Production in Medaka.** In addition to the effects observed in cells, we further examined the responses of medaka fish upon FSK exposure. The transcriptional levels of the genes involved in steroidogenesis were firstly examined, and significant up-regulations were observed for six genes, including *HMGR*, *HSD3B2*, *CYP17B*, *CYP17B1*, *CYP19A*, and *CYP21A*. Among them, *HMGR* was the most significantly affected. The transcriptional levels were markedly induced by 35.3, 58.9, and 44.0 times (fold change) in medaka upon exposure to 0.3 μg/L, 3 μg/L, and 30 μg/L FSK, respectively. This was followed by *CYP19A*. *CYP19A* exhibited induction in transcriptional levels by 27.0 and 22.7 times in medaka upon exposure to 3 μg/L and 30 μg/L FSK, respectively. In addition, *CYP17* and *CYP21A* were also up-regulated by 9.9, 7.6, and 6.2 times, and 0.2, 5.6, and 5.8 times in medaka upon 0.3 μg/L, 3 μg/L, and 30 μg/L FSK exposure, respectively (Figure 3). This was accompanied by alterations in steroid concentrations. We measured the 17β-E_2_ and ADD levels in medaka and found that 17β-E_2_ was remarkably up-regulated by 3.2, 3.2, 9.0, and 4.1 times and ADD was slightly down-regulated by 3.2, 3.2, 9.0, and 4.1 times in medaka upon 0.03 μg/L, 0.3 μg/L, 3 μg/L, and 30 μg/L FSK exposure, respectively. However, no statistically significant differences were observed for steroid hormone levels. This phenomenon was possibly due to significant individual differences and the limited replicate number (*n* = 6) of the fish samples, as described in Figure 3 and Table S5.

**Figure 4:**
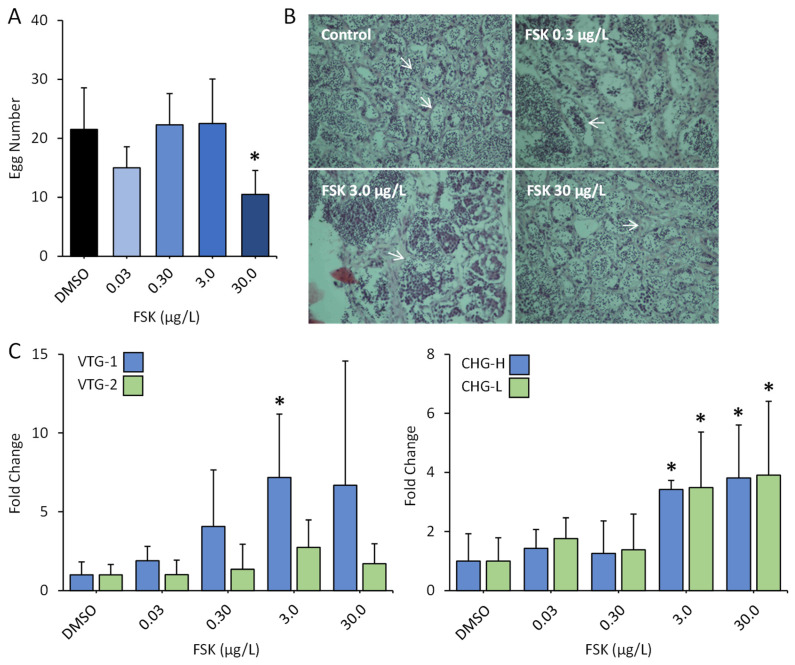
Evaluation of endocrine-disruptive effects of forskolin in medaka. (**A**) Mean egg numbers of medaka in control and forskolin-exposed groups. (**B**) Light micrographs of the testes in control group and forskolin-exposed groups. (**C**) Fold changes in biomarker genes for estrogenicity including *VTG-I*, *VTG-II*, *CHG-H*, and *CHG-L* in livers of male medaka in control and forskolin-exposed groups (*n* = 6). Asterisk (*) indicates statistically significant differences from the control (*p* < 0.05).

**Reproductive and Developmental Effects.** The reproductive and developmental effects of FSK in exposed medaka fish were further examined. As shown in Figure 4 and Figures S1–S3, we observed a significant decrease (by 65%) in reproductive success (egg numbers) in medaka upon 30 μg/L FSK exposure. Meanwhile, negligible effects were observed for the rate of spawning, the sex ratio (F/M: female/male), and the fertilization success of the F1 eggs of medaka. Furthermore, histopathological examination was performed for the gonads of male medaka with H&E staining, revealing thickening of the lumen wall and inhibition of spermatogenesis. The proportion of immature sperm significantly increased (Figure 4B). The biomarker genes associated with estrogenicity [31,32], including *VTG-I*, *VTG-II*, *CHG-L*, and *CHG-H*, were tested in the livers of male medaka. As shown in Figure 4C, *VTG-I*, *CHG-L*, and *CHG-H* all displayed significant alterations. Particularly, the transcriptional levels of *CHG-L* and *CHG-H* were markedly induced by 3.5 and 3.9 times, and 3.4 and 3.8 times in medaka upon exposure to 3 μg/L and 30 μg/L FSK, respectively. *VTG* and *CHG* serve as precursors of egg yolk protein and inner layer subunits of the egg envelope, respectively [33,34]. Their synthesis in male oviparous vertebrates are recognized as sensitive biomarkers of estrogenic pollutants [31,32]. The alterations in *VTG* and *CHG* levels, along with the observed histopathological and reproductive effects, clearly revealed the endocrine-disruptive effect of FSK in organisms.

As an adenylate cyclase activator, FSK may disrupt *CYPs*’ activities by activating cAMP-dependent protein kinase [35], thereby leading to disturbances in steroidogenesis homeostasis. In the present study, we systematically tested and confirmed this hypothesis with in vitro and in vivo experiments. It should be noted that the effects of forskolin may not be solely attributed to its endocrine-disrupting properties but could also be linked to its teratogenic effects during the early development of medaka. As a model organism, the reproductive and developmental effects observed in medaka may also have broader implications for other organisms. Meanwhile, considering FSK’s broad use as a pharmaceutical agent in humans and possible introduction into aquatic environments through WWTP effluents, concerns arise regarding its potential adverse effects in both humans and diverse aquatic animals. This warrants increased attention and further investigation.

## 4. Conclusions

By the use of the in vitro H295R steroidogenesis assay and the in vivo long-term medaka exposure assay, we systematically examined the potential endocrine-disruptive effects of FSK. Our results demonstrated that the transcriptional levels of a series of genes involved in steroidogenesis and the production of steroids were remarkably altered in H295R cells. Additionally, the results were consistent with the outcomes obtained from the medaka after FSK waterborne exposure. In addition, significantly induced biomarker genes for estrogenicity, pathological damage to the gonads, and decreased reproductive success were further revealed. Thus, the results demonstrated that FSK modulates the transcriptions of steroidogenesis genes and alters hormone levels in vitro and in vivo, which in turn lead to endocrine disruption in organisms. The present analysis provides a novel perspective into understanding the toxicological effects of FSK, thereby contributing new insights to investigate EDCs.

## Data Availability

The data presented in this study are available on request from the corresponding author.

## Supplementary Materials

The following supporting information can be downloaded at https://www.mdpi.com/article/10.3390/toxics12100701/s1: Table S1: Primer sequences for quantitative real-time PCR analysis in H295R cells; Table S2: Primer sequences for quantitative real-time PCR analysis in medaka; Table S3: Fold changes in expressions of eleven genes relating to steroidogenesis in H295R cells exposed to forskolin; Table S4: Fold changes in production of eight steroid hormones in H295R cells exposed to forskolin for 48 h; Table S5: Fold changes in expression of 14 genes relating to steroidogenesis in gonad of male medaka exposed to forskolin for 100 days; Figure S1: Rate of spawning of medaka and fertilization success of F1 eggs in control and forskolin-exposed groups; Figure S2: Sex ratio (F/M: female/male) of medaka in control and forskolin-exposed groups; Figure S3: Hepatic-somatic index (HSI) and gonadal-somatic index (GSI) of medaka in control and forskolin-exposed groups.
