# Supplementary material for: Endocrine-Disruptive Effects of Adenylate Cyclase Activator Forskolin: In Vitro and In Vivo Evidence
Source: Toxics. 2024 Sep 27;12(10):701. doi: 10.3390/toxics12100701 (PMC11510926; doi:10.3390/toxics12100701)

Supplementary Material

Endocrine-Disruptive Effects of Adenylate Cyclase Activator Forskolin: In Vitro and In Vivo Evidence

Chong Huang [1], Yanbin Zhao [1,2,*] and Jianying Hu [1]

[1] MOE Laboratory for Earth Surface Processes, College of Urban and Environmental Sciences, Peking University, Beijing 100871, China; hc@urban.pku.edu.cn (C.H.); hujy@urban.pku.edu.cn (J.H.)
[2] State Environmental Protection Key Laboratory of Environmental Health Impact Assessment of Emerging Contaminants, School of Environmental Science and Engineering, Shanghai Jiao Tong University, Shanghai 200240, China
* Correspondence: zhaoyanbin@sjtu.edu.cn; Tel.: +86-188-1820-7732

Number of pages: 9

Number of figures: 3

Number of tables: 5

Table S1. Primer sequences for quantitative real-time PCR analysis in H295R cells. For the primers designed, the intron/exon boundary-spanning primers were preference to minimize the genomic DNA contamination. Primer efficiency values are ranging from 90% to 110%.

Gene	Accession No.	Sequence	Amplicon Size (bp)
HSD3B1	NM000862	5′-AGCATCTTCTGTTTCCTGGTG-3′	141
		5′-TCTCCTTCAGCTCCTTCTCCTT-3′	
HSD3B2	NM000198	5′-AGCATCTTCTGTTTCCTGGCA-3′	142
		5′-TCTCCTTCAGTTCCTTCTCTTC-3′	
HSD17B1	NM000413	5′-TTCATGGAGAAGGTGTTGG-3′	104
		5′-AAGACTTGCTTGCTGTGG-3′	
CYP11A	NM000781	5′-GAGATGGCACGCAACCTGAAG-3′	137
		5′-CTTAGTGTCTCCTTGATGCTGGC-3′	
CYP11B1	NM000497	5′-GGTTTGCCAGGCTAAGC-3′	113
		5′-CAAACTGCCCAGAGGACAG-3′	
CYP11B2	NM000498	5′-TCCAGGTGTGTTCAGTAGTTCC-3′	146
		5′-GAAGCCATCTCTGAGGTCTGTG-3′	
CYP17	M14564	5′-GGCACCAAGACTACAGTGATTG-3′	147
		5′-AGAGTCAGCGAAGGCGATAC-3′	
CYP19	NM000103	5′-TTGGAAATGCTGAACCCGATAC-3′	93
		5′-GCCAGTGAGGAGCAGGAC-3′	
CYP21	NM000500	5′-ACCTCAGTTTCTCCTTTATTGC-3′	94
		5′-AGAGCCAGGGTCCTTCAC-3′	
HMGR	NM000859	5′-TTCAGGTTCCAATGGCAACAAC-3′	122
		5′-GCCACGAGTCATCCCATCTG-3′	
StAR	NM000349	5′-ATGAGTAAAGTGGTCCCAGATG-3′	143
		5′-ACCTTGATCTCCTTGACATTGG-3′	
β-actin	BC002409	5´-AAACTACCTTCAACTCCATC3´	163
		5´-ATGATCTTGATCTTCATTGT-3´	

Table S2. Primer sequences for quantitative real-time PCR analysis in medaka. For the primers designed, the intron/exon boundary-spanning primers were preference to minimize the genomic DNA contamination. Primer efficiency values are ranging from 90% to 110%.

Gene	Accession No.	Sequence	Amplicon Size (bp)
RPL-7	DQ118296	5'- CGCCAGATCTTCAACGGTGTAT -3'<br>5'- AGGCTCAGCAATCCTCAGCAT -3'	72
VTG-I	AB064320	5'-CTCCAGCTTTGAGGCCATTTAC-3'<br>5'-ACAGCACGGACAGTGACAACA-3'	81
VTG-II	AB074891	5'- CCAAGACCAAAGACCTGAACC-3'<br>5'- TAAGATTAGGGAACCAGTAGT -3'	167
CHG-H	AF500195	5'- TACTTTCCCGTCACTTATTGC-3'<br>5'- TTCCACGACCAGAGTTTCAAC -3'	189
CHG-L	AF500194	5'- CAACATCTGCTGCTTATCCCC-3'<br>5'- GACATCGCCTTCCCATTCCAG-3'	257
CYP19A	D82968	5'-GCGTAGAGCCCTTTTCGATGA-3'<br>5'-TGCGGCCCGTATTCAAGAT-3'	80
CYP17B	D87122	5'-CCCCTGGTTACAGTCAGACTACAG -3'<br>5'- CAGCAGGGCGTCCAGAAG -3'	61
CYP17A	D87121	5'- CCCCTGGTTACAGATTTTTCCC -3'<br>5'-TGCAGCAGCTGGTCTCTAACTG -3'	78
StAR	DQ988930	5'- GAAGCAAGGCGAAGATGCAC -3'<br>5'- TTTCAACAGTCCAGCCGTCC -3'	71
HSD17B1	EF530597	5'-CTTGGCTGGAATGAAAGCACA -3'<br>5'-TGAAAGGAAGCCCATGGAGTC -3'	80
HSD17B3	EF530598	5'- TCTTATACAGGCAGTGGCTCCA-3'<br>5'-GGTAAAAAGGTCACCCTGTTGG -3'	81
CYP11A	EF537029	5'- TTGCCGTGAGTCTGCAAAGATA-3'<br>5'- AAAGTCCCAGCCGGGATGT-3'	73
HSD11B2	EF537022	5'-CCTCAGAGGTCTGTGGGGTTTAG -3'<br>5'-TGGGCTGGAGATGGTGATAATG -3'	190
HSD20B	EF537021	5'-GATGTGGACAGCATCAGCACTG-3'<br>5'-AGTCGTGTCTGCCACCTTGAAC-3'	108
CYP21A	EF537024	5'-GCTCATCACGATCCTGCAGTCT -3'<br>5'- AGCGCTCTGGTTTGAAGCTGT-3'	55
HSD3B1	EF537026	5'- CTGTCGAGGTGCAACAAACGT-3'<br>5'- CACCGACTCCAGCACATCAAT-3'	61
HSD3B2	EF537025	5'- CCGACCACTACTCCAGAACCAA-3'<br>5'- TCTTTCAGCACTGTCCCGTTG -3'	73
HMG-CoA	EF537031	5'- TGATGGCTTTCAGGCAGTCA-3'<br>5'- GCAAGTCCAACCAGCAACCT-3'	81

Table S3. Fold changes in expressions of eleven genes relating to steroidogenesis in H295R cells exposed to forskolin. Data are expressed as fold change relative to the solvent control (n = 3). Asterisk* indicates statistically different from control ($p < 0.05$).

Gene	DMSO	Concentration of Forskolin			
		0.3 μM	1 μM	3 μM	30 μM
Star	1.0±0.3	1.3±0.3	1.1±0.3	1.1±0.4	1.7±0.3*
HMGR	1.0±0.2	1.2±0.4	1.0±0.1	1.0±0.7	1.5±0.1
CYP11A	1.0±0.1	1.6±0.3*	0.9±0.2	2.1±0.2*	1.9±0.1*
CYP21	1.0±0.2	5.0±0.9*	5.2±1.0*	2.8±1.0*	3.9±0.7*
HSD3B1	1.0±1.0	0.8±0.1	0.7±0.2	1.2±0.6	1.3±0.1
HSD3B2	1.0±0.1	20.7±5.7*	23.2±12.8*	15.6±9.5*	20.5±4.1*
HSD17B1	1.0±0.4	1.1±0.0	1.3±0.7	2.6±1.9*	1.1±0.3
CYP17	1.0±0.4	2.4±0.8*	1.9±0.3	3.6±1.2*	3.1±0.5*
CYP19	1.0±0.7	0.5±0.3	0.6±0.3	1.7±0.7	4.1±2.4*
CYP11B1	1.0±0.6	1.7±0.3	1.3±0.1	6.5±8.4	2.0±0.5
CYP11B2	1.0±0.5	2.6±0.9	2.6±0.5	8.8±2.2*	9.0±1.5*

Table S4. Fold changes in production of eight steroid hormones in H295R cells exposed to forskolin for 48 hours. Data are expressed as fold change relative to the solvent control (n = 3). Asterisk* indicates statistically different from control ($p < 0.05$).

Hormone	DMSO	Concentration of Forskolin			
		0.3 μM	1 μM	3 μM	30 μM
TTR	1.0±0.1	0.6±0.1*	0.6±0.2*	0.5±0.1*	0.6±0.1*
ADD	1.0±0.1	0.6±0.1*	0.7±0.2*	0.6±0.1*	0.7±0.2*
PGT	1.0±0.1	1.3±0.1	1.5±0.0*	1.6±0.1*	2.1±0.2*
17-HPT	1.0±0.1	2.9±0.2*	3.1±0.4*	2.8±0.1*	3.2±0.1*
CRL	1.0±0.1	0.9±0.3	1.1±0.2	1.2±0.2	1.2±0.2
CRT	1.0±0.2	1.1±0.3	1.0±0.1	0.9±0.1	1.0±0.1
17β-E2	1.0±0.1	14.1±1.1*	18.4±3.4*	20.6±2.1*	15.1±4.4*
E1	1.0±0.1	22.9±2.7*	31.6±6.4*	27.0±2.6*	19.5±4.5*

Table S5. Fold changes in expression of 14 genes relating to steroidogenesis in gonad of male medaka exposed to forskolin for 100 days (n = 6). Asterisks (*) indicates statistically significant difference from the control ($p < 0.05$).

Gene	DMSO	Concentration of Forskolin			
		0.03 μg/L	0.3 μg/L	3 μg/L	30 μg/L
Star	1.0±0.3	0.7±0.0	0.8±0.1	1.0±0.3	0.6±0.2
HMGR	1.0±0.4	0.8±0.3	5.3±31.4*	8.9±13.5*	44.0±5.2*
CYP11A	1.0±0.7	0.8±0.4	0.9±0.3	0.9±0.3	0.7±0.4
CYP21A	1.0±2.3	0.4±0.8	0.2±0.3	5.6±10.0*	5.8±3.8*
HSD3B1	1.0±0.1	1.0±0.1	1.9±0.9	1.3±0.4	0.9±0.4
HSD3B2	1.0±0.1	1.4±0.1	1.3±0.6*	1.3±0.2	1.0±0.3
HSD20B	1.0±0.1	1.2±0.3	1.0±0.4	1.1±0.4	1.1±0.1
HSD17B1	1.0±0.5	0.6±0.2	0.7±0.4	4.7±4.3	5.5±1.7*
HSD11B2	1.0±0.6	1.0±0.3	1.0±0.2	0.6±0.1	0.6±0.1
HSD17B3	1.0±0.1	1.4±0.3	1.4±0.7	0.9±0.2	0.9±0.2
CYP17A	1.0±0.4	0.7±0.2	1.1±0.4	0.9±1.3	0.7±1.1
CYP17B	1.0±0.5	1.5±0.3	9.9±8.7*	7.6±2.0*	6.2±2.9

Figure S1. Rate of spawning of medaka and fertilization success of F1 eggs in control and forskolin exposed groups. Asterisks (*) indicates statistically significant difference from the control ($p < 0.05$).

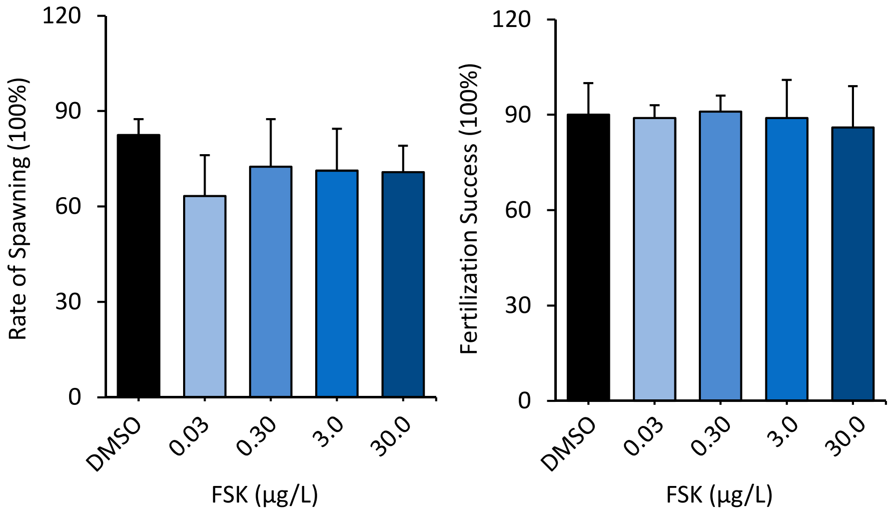

Figure S2. Sex ratio (F/M: female/male) of medaka in control and forskolin exposed groups. Asterisks (*) indicates statistically significant difference from the control ($p < 0.05$).

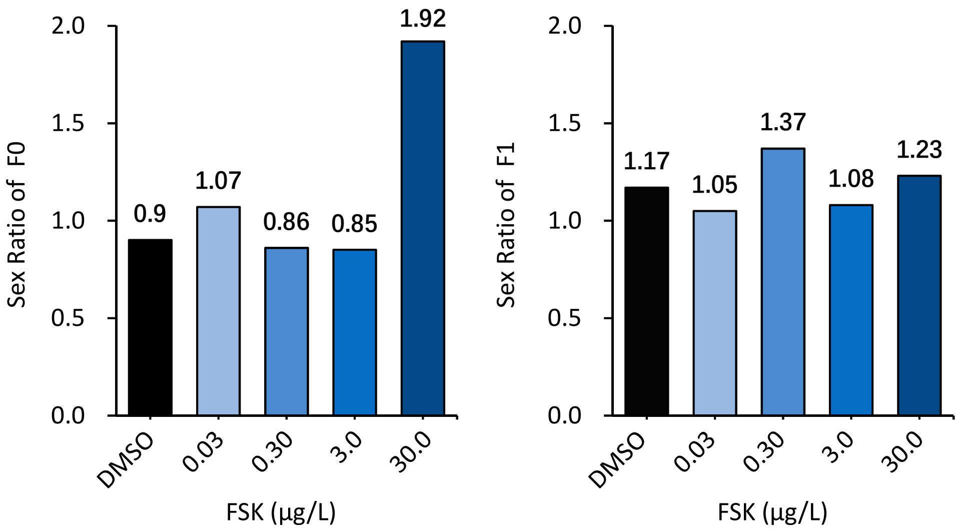

Figure S3. Hepatic-somatic index (HSI) and gonadal-somatic index (GSI) of medaka in control and forskolin exposed groups (n = 6). Asterisks (*) indicates statistically significant difference from the control ($p < 0.05$).

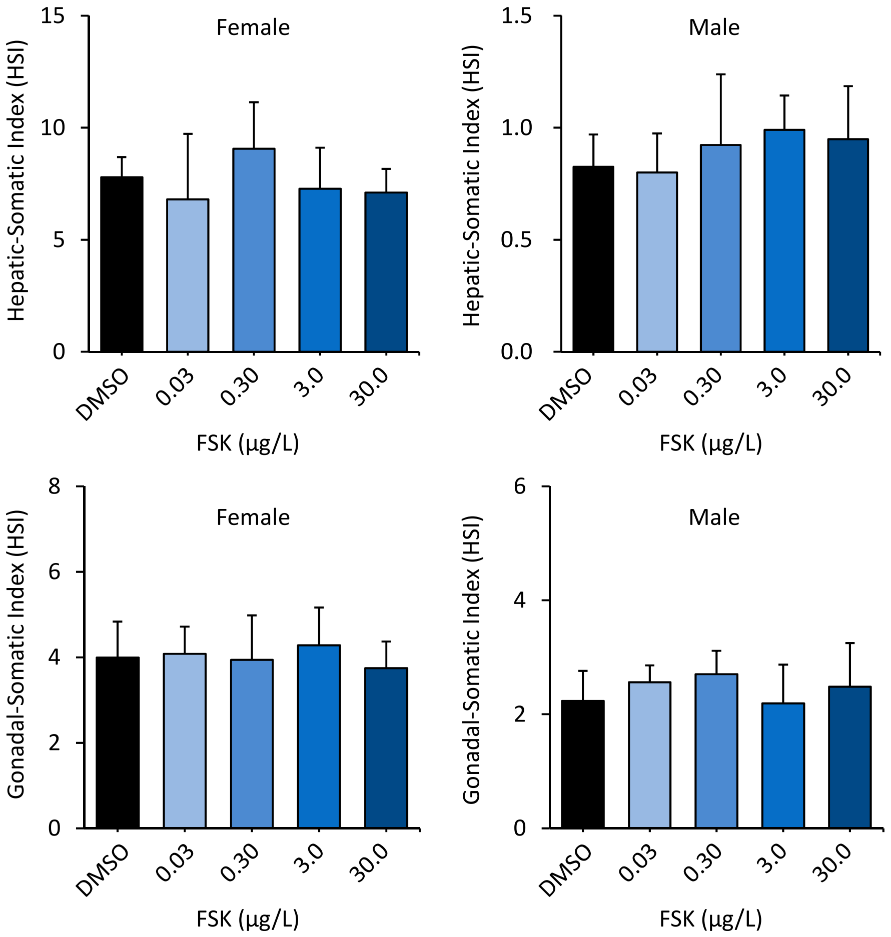

Supplement: Supplementary file 1 [file toxics-12-00701-s001.zip › toxics-3204142-supplementary.pdf]
